# Retraction: Pathogenesis of malignant pleural mesothelioma and the role of environmental and genetic factors

**DOI:** 10.1186/1477-3163-7-4

**Published:** 2008-08-08

**Authors:** Shoshana J Weiner, Siyamek Neragi-Miandoab

**Affiliations:** 1 Cleveland Clinic Lerner College of Medicine, Case Western Reserve University, 9500 Euclid Avenue, Cleveland, OH, USA; 2 University Hospitals, Case Western Reserve University School of Medicine, 11100 Euclid Avenue LKS Building 7^th ^floor, Cleveland, OH, USA

BioMed Central is publishing a retraction of this article [1] as the article was published in error. BioMed Central apologize to the authors for the error and any inconvenience caused. An apology is also extended to the readers.
